# Does the Formation of a Taylor Cone in a Pulsating Electrospray Directly Impact Mass Spectrometry Signals?

**DOI:** 10.1021/acsomega.4c07653

**Published:** 2024-10-11

**Authors:** Ching-Han Chang, Pawel L. Urban

**Affiliations:** Department of Chemistry, National Tsing Hua University, Hsinchu 300044, Taiwan

## Abstract

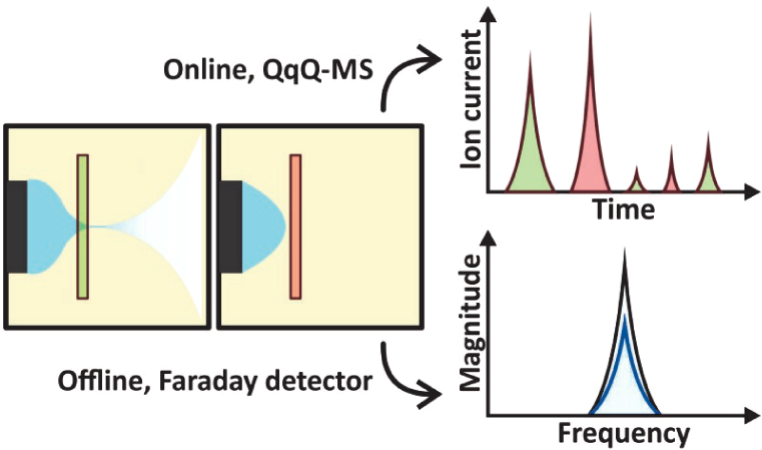

Electrospray ionization (ESI) remains the dominant technique in mass spectrometry (MS)-based analyses. Here, we investigated the relationship between a crucial aspect of ESI, the formation of the Taylor cone, and the MS ion current by utilizing a triple quadrupole (QqQ) mass spectrometer coupled with a streaming high-speed camera and a 3-ring electrode system. In one test, ion current over a 30-s plume gate (a ring electrode) opening was compared with the Taylor cone occurrence analyzed offline, with Spearman’s correlation coefficients consistently near 0 despite parameter variations. In another test, real-time detection of Taylor cones was synchronized with QqQ-MS, selectively opening (de-energizing) the plume gate based on the Taylor cone status. This approach enabled matching the ion current with the Taylor cone occurrence. There was no apparent difference between the MS signals recorded in the presence and absence of a Taylor cone. Additionally, a Faraday plate was employed as a detector in offline experiments, revealing agreement between the frequency of liquid meniscus (Taylor cone) oscillation (∼1.92 kHz)—measured by high-speed imaging—and the frequency of spray current (∼1.93 kHz). We suggest that the lack of positive correlations in the MS experiments is due to intrinsic ion carryover during transit from the ion source to the detector and due to the insufficient data acquisition rate of the mass spectrometer, which erases short-term fluctuations of ion current.

## Introduction

Electrospray ionization (ESI) mass spectrometry (MS) has revolutionized the analysis of biomolecules and nonvolatile or thermolabile compounds by offering distinct advantages.^1−8^ These include its compatibility with liquid chromatography (LC), reduced occurrence of ion fragmentations, and ability to produce multiply charged ions,^1,2^ which enables accurate and comprehensive chemical analysis.^7^ A high potential difference between the ESI emitter and the counter electrode causes the spray phenomenon.^9^ Under a strong electric field, the interaction between liquid surface tension and surface charge repulsion forces the emission of polydisperse clouds of charged droplets from the apex of a characteristic feature called the Taylor cone.^10−12^ The charged droplets subsequently experience solvent evaporation and then contract to a specific size, at which point Coulombic jet fission takes place, producing smaller progeny droplets.^13^ The process of evaporation and fission repeats several times, yielding nanoscale droplets.^12,14^ As the charge density increases, those nanodroplets reach the Rayleigh limit, transferring charges to the analyte or breaking up into tiny progeny droplets.^15^ The release of gaseous ions occurs through various mechanisms,^16^ including the ion evaporation model,^17−19^ charged residue model,^20^ and chain ejection model.^16,21^

The electrospray spraying modes can be controlled by adjusting experimental parameters such as the strength of the electric field, emitter dimensions, liquid flow rate, and solvent physical properties. Based on a given liquid flow rate, the spraying modes—listed in the increasing order of applied voltage—include dripping, microdripping, spindle, multispindle, steady cone-jet, oscillating jet modes, precession mode, ramified jet mode, rim emission mode, and multijet mode.^22−25^ Various spraying modes yield different droplet sizes. Among these modes, the steady cone-jet mode stands out for its exceptional stability and monodisperse micro- or nanodroplet production, resulting in high ionization efficiency.^26−28^ Despite the ongoing efforts to characterize electrospray spraying modes—dating back to the 20th century^9,29^—a comprehensive explanation of the ESI mechanism remains elusive. Some of the reasons are the inherent pulsations in electrospray (∼1–2 kHz) in the self-oscillating electrospray mode,^11,30^ the high velocity, and the minuscule size of the droplets.

Previous reports suggest that the primary droplets with larger volumes exhibit a propensity to concentrate along the central axis of the ESI plume. Conversely, smaller satellite and progeny droplets are more likely to be repelled toward the plume periphery.^29−31^ However, the larger droplets (>10 μm)—undergoing more repeated fission cycles—fail to efficiently produce gaseous analyte ions,^32^ whereas smaller nanodroplets facilitate efficient ion formation and enhance MS signals.^12,33,34^ The ESI efficiency hinges on the initial droplet size, determined by solvent composition,^35,36^ liquid flow rate,^37^ and emitter tip diameter.^38−40^ This spatial distribution of electrospray droplets results in lower ionization efficiency of ESI due to substantial analyte wastage before entering the MS inlet.^34,40^ Nano-ESI is another choice featuring a micrometer-scale inner diameter of the emitter.^38,41^ Its extensive use in MS studies is attributed to outstanding ionization efficiency, reaching approximately 100% at sufficiently low flow rates, and the advantageous feature of low sample consumption measured in nanoliters per minute.^38,42^ Nonetheless, challenges arise from the susceptibility of the nano-ESI emitter to clogging and its low reproducibility when relying on manual fabrication.

Various techniques have been used to investigate the droplet size, velocity, and charges in electrospray.^43−46^ As one of the most direct methods, photography-based visualization is employed to investigate the characteristics of charged droplets in the presence of an electric field.^47−49^ In recent years, high-speed imaging has gained prominence in electrospray droplet studies, owing to its capability for sensitive visualization at high speeds. This technique proves formidable, addressing considerations such as the droplet size, trajectory, electrospray spraying mode, and vapor formation.^32,50−54^ Despite the significant benefits associated with high-speed imaging techniques, the fast motion of droplets presents an obstacle, hindering real-time, high-speed analysis in ESI-MS research.

In the domain of artificial intelligence, computer vision (CV) focuses on enabling computers and systems to extract meaningful information from digital images, videos, and other visual inputs. This capability allows the systems to take action or provide recommendations based on the acquired information.^55^ CV has been employed in food chemistry,^56−58^ organic synthesis monitoring,^59^ and fluid dynamics.^60^ By utilizing CV, real-time analysis has been applied to MS studies such as the quality control of LC-MS and the feedback control of the ESI voltage.^61,62^ The advantages of using CV include automatic detection, convenience,^63^ simplicity, speed, reliability, accuracy, and reduction of costs.^64^ A streaming high-speed camera can be used to achieve an extremely rapid real-time analysis of the process of ESI-MS.

In this work, we aimed to precisely manipulate the ESI plume in accordance with experimental demands by integrating ESI-MS with electronically controlled ring electrodes (REs). Furthermore, real-time monitoring of the ESI emitter tip using a high-speed camera (HSC) allowed for visual observation of the Taylor cone formation, facilitating subsequent offline analysis postexperimentation. Moreover, by integrating the three RE system with the HSC for real-time imaging, we achieved instantaneous analysis in ESI-MS and enabled the selection of the electrospray output generated by a single liquid meniscus (Taylor cone) oscillation cycle via real-time control (RTC).

## Experimental Section

### Chemicals

Acetaminophen (meets USP testing specs, 98.0–102.0%) was purchased from Sigma-Aldrich (St. Louis, MO, USA). It was dissolved in a mixture of methanol (LC-MS grade) and water (LC-MS grade), which were purchased from Fisher Scientific (Waltham, MA, USA).

### ESI-RE-MS Setup

**Figure 1:**
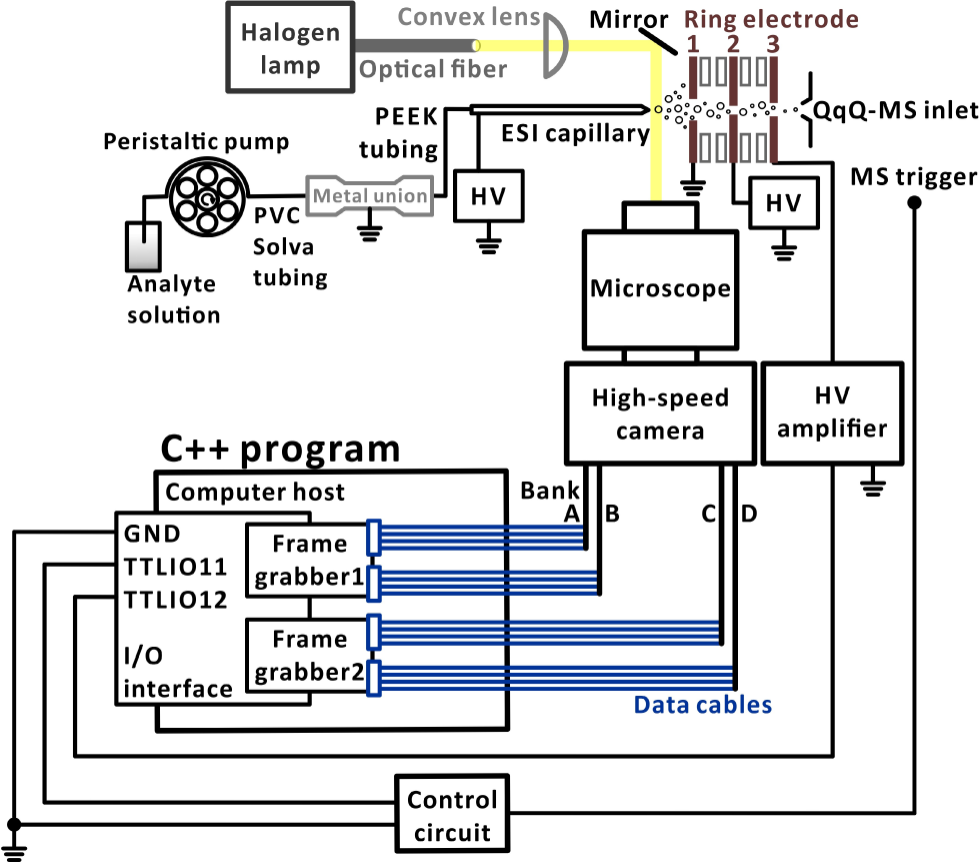
Simplified scheme of the ESI-RE-MS setup used in the online experiments.

The experimental setup (Figure 1)—used in most experiments—has been adapted from the previous work.^32^ In essence, it comprises an electrospray capillary (I.D., 100 μm; O.D., 270 μm; length, 82.5 mm; part no., 225–14915; Shimadzu, Kyoto, Japan) and three concentric REs (width, 40 mm; height, 40 mm; thickness, 0.8 mm; part no., 91587; product no., CGS-1015–0.8-Single; material, Bakelite with copper coated on one side; Centenary Materials, Hsinchu, Taiwan) positioned in front of the ESI capillary. The orifice diameters of the REs are 10 mm, 5 mm, and 7.5 mm, respectively. The metal capillary was firmly secured within a 3D-printed holder (core material, acrylonitrile-butadiene-styrene; Tiertime, Beijing, China). The REs were separated by two insulating spacers (width, 40 mm; height, 40 mm; thickness, 1 mm; part no., 130680; material, polytetrafluoroethylene (PTFE); Centenary Materials, Hsinchu, Taiwan) between each ring electrode. This ESI-RE setup was coupled with a triple quadrupole mass spectrometer (QqQ-MS; LCMS-8030; Shimadzu). Maintaining a distance of 7 mm between the ESI capillary tip and the RE1 and 5 mm between the RE3 and the sampling cone of QqQ-MS ensures optimal performance. Additionally, the height of the ESI capillary is elevated by ∼1 mm relative to the MS inlet to prevent direct interference with the drying gas flow.

### Sample Injection Setup

The peristaltic pump (ISM4412; Ismatec, Wertheim, Germany) was used to infuse the liquid sample through transfer tubings at a flow rate of 15 μL min^–1^. The first section of the flow line is composed of two tubings made of different materials: a poly(ether ether ketone) (PEEK) tubing (I.D., 0.13 mm; O.D., 1.59 mm; length, 10 cm; GL Sciences, Tokyo, Japan) and a PVC Solva tubing (I.D., 0.13 mm; O.D., 2.05 mm; length, 42 cm; part no., 168–001A-101; Ismatec). The immersed PVC Solva section effectively interfaces with the liquid sample, while the PEEK tubing is linked to one side of the grounded metal union (I.D., 0.5 mm; O.D., 1.5875 mm; thread port configuration, 10–32 coned; part no., U-412; material, 316 stainless steel; IDEX Health and Science, Rohnert Park, CA, USA). The second PEEK tubing (I.D., 0.13 mm; O.D., 1.59 mm; length, 40 cm; GL Sciences, Tokyo, Japan) is affixed at one end to the ESI capillary and—at the other end—to the opposing side of the grounded metal union.

### High-Speed Imaging Setup

Images were captured through a streaming machine-vision high-speed camera (S710; Phantom, Wayne, NJ, USA) configured with a resolution of 256 × 256 pixels and an exposure time of 1 μs. For electrospray shadowgraph acquisition, a stereomicroscope (SMZ745T; Nikon, Tokyo, Japan) was affixed to the HSC and the camera was oriented orthogonally to the ESI capillary. Intense light for illumination was provided by a halogen lamp with an attached optical fiber (OSL2IR; Thorlabs, Newton, NJ, USA), placed next to the ESI capillary. To enhance the light beam’s focus, a convex lens (diameter, 40 mm; focal length, 65 mm; material, glass; Tiger, Taoyuan, Taiwan) was installed in front of the optical fiber, directing the light to a 45°-angled mirror (diameter, 22 mm; material, glass; Yifu Hardware, Taoyuan, Taiwan). This mirror reflected the light onto the ESI capillary, stereomicroscope, and HSC. Two frame grabbers (Coaxlink Octo; Euresys, Liège, Belgium)—linked by 16 data cables (CX-34–1–34–05 CoaXPress Cables; Components Express, Woodridge, IL, USA) and inserted into the PCIe3 interface of a computer host (ASUS W980T workstation, Taipei, Taiwan)—were used to control the HSC. Each set of four cables, denoted as bank A, bank B, bank C, and bank D, respectively, played a distinct role in image acquisition. In the continuous image acquisition system, the HSC with four banks was triggered by an external I/O interface (HD26F 3304 external I/O connector; Euresys), and the control was performed by a microcontroller board (Arduino Due, Centenary Materials). By contrast, in the RTC system driven by a C++ program (see the Supporting Information), only bank A transmitted buffer information through four parallel cables, while the other banks remained disconnected. The program was written using the eGrabber programmer guide^65^ as a reference. ChatGPT (OpenAI, San Francisco, CA, USA) was used for debugging.

### Real-Time Image Analysis

**Figure 2:**
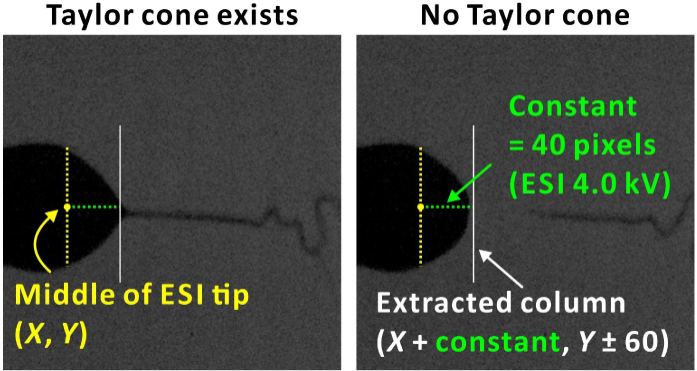
Representative photos of the electrospray emitter with and without Taylor cone. Each image was analyzed individually. The user manually selected the middle of the ESI emitter tip (*X*, *Y*; yellow point) when the image was read. The algorithm then created a reference line (white line, 120 pixels) in front of the ESI emitter tip. The horizontal offset values were constant for each ESI voltage. For instance, the horizontal offset was 40 pixels when the ESI voltage was set to 4.0 kV. These offset values were determined from high-speed imaging results and were fixed throughout the entire image processing procedure.

To construct the RTC system, the HSC was triggered through eGrabber (C++ standard library; Euresys). At the same time, the RE3 voltage and MS acquisition were controlled via an external I/O interface connected to the frame grabber. A real-time analysis loop was established in a C++ program for control of the RE3 (Figure S1A). In each real-time analysis cycle, the HSC monitored the tip of the ESI capillary, capturing a buffer for subsequent pixel analysis. During pixel analysis, the buffer was read from RAM, and then, a column of 120 rows was extracted to provide information on the existence of the Taylor cone (Figure 2). Following each cycle of real-time image analysis, the frame grabber triggered the RE3 (acting as an electrospray plume gate) to open (0 V) or close (3.3 V) based on specific conditions through pin 17 (TTLIO12) of the I/O interface.

### Electronic Circuit for Control System

The formation of electrospray involved the application of high voltage (HV) from an HV power supply (MPS10P10; Spellman, Hauppauge, NY, USA) to the ESI capillary. The RE1 was grounded; the RE2 was connected to an independent HV power supply (MPS10P10; Spellman) set to 300 V; and the RE3 was connected to an HV amplifier (gain, 500; TREK 609A–1; Advanced Energy, Denver, CO, USA). The reason for energizing the RE2 is to focus on the electrospray plume.^32,66^ The external I/O interface served as the central processor for the control circuit. To synchronize data acquisition between MS and HSC data, a 3.3 V pulse was sent to MS trigger ports through pin 25 (TTLIO11) of the I/O interface, facilitated by a relay board (model no. 2R1B; Centenary Materials, Hsinchu, Taiwan). The provision of asymmetric square waveforms (*V*_pp_ = 1650 V) to the RE3 was accomplished by connecting the HV amplifier to pin 17 (TTLIO12), supplying 3.3 V.

### Mass Spectrometry

The analyses were performed on the QqQ-MS instrument in which a homemade ESI setup replaced the commercial ESI source. The desolvation line (ion-transfer tube) temperature was 250 °C; the heat block temperature was 400 °C; and the drying gas flow rate was 3.0 L min^–1^. All the analyses were carried out in positive-ion selected-ion-monitoring (SIM) mode at *m*/*z* 152. During each acquisition event, dwell time was 0.8 ms and event time was 0.004 s, which means it took 0.8 ms to detect ions at the set *m*/*z* and 4 ms to get one intensity value. These are the smallest time settings available in the instrument used here. The choice of SIM mode and monitoring a single analyte was to maximize the rate of data acquisition. The voltage of the commercial ion source was set to 0.00 V to prevent its influence on the current custom-built setup.

### Electronic Circuit for Offline Setup

In an offline experiment, we employed a more straightforward configuration to record the spray current (Figure S2). A homemade Faraday plate (diameter, 22 mm; thickness, 2 mm; material, aluminum; Scientific Instrument Center of the National Tsing Hua University, Hsinchu, Taiwan) was connected to the oscilloscope (SDS6062; OWON Technology, Zhangzhou, China) via an SMA-KFD connector (part no., 124822; Centenary Materials, Hsinchu, Taiwan), an SMA-BNC connector (part no., 3330; Centenary, Hsinchu, Taiwan), and a BNC cable (part no., 157077; Centenary, Hsinchu, Taiwan). The current was sampled at a rate of 20 μs. The electrospray image capturing was carried out at a frame rate of 50 kfps by the HSC.

### Data Treatment

In the first experiment, all images were captured by the HSC and then sent to eGrabber Studio software for temporary storage. Every 100 buffers were merged into one image, achieving a total of 1500 images at a frame rate of 5000 fps, totaling 150 000 frames. Subsequently, a Python program was utilized to extract 150 000 frames from the 1500 images, which were then imported into MATLAB (version R2022b; MathWorks, Natick, MA, USA) for offline image analysis (Figure S1B). During the image analysis, the reference line (length, 120 pixels) was set at a distance of 40 pixels from the ESI emitter (Figure 2), and the existence of the Taylor cone was determined based on the number of black pixels (within the reference line) by a MATLAB program (see the Supporting Information). Ultimately, 150 000 data points were collected. These data were then analyzed by calculating the percentage of Taylor cones for every 20 data points by another MATLAB program (see the Supporting Information). A Spearman’s correlation analysis was then performed for 7500 MS signals. Considering that in the experimental process, images were obtained before MS received signals, and for more reliable data processing results, we also adjusted the time shift in increments of 0.2 ms to match the occurrence of ion currents with Taylor cones.

In the second experiment, we employed real-time detection of Taylor cones and relayed the ESI output to the mass spectrometer by opening (de-energizing) the plume gate (RE3) only under a specific condition, when the Taylor cone was either present or absent. Due to the elongation of the Taylor cone shape under lower ESI voltage, the distances of the reference lines from the emitter tip were different (30–50 pixels) and were selected for various ESI voltages. This experiment enabled the matching of the ion currents with the occurrence of Taylor cones.

In the third experiment, high-speed images were captured simultaneously with the ESI spray current. These images were analyzed using the same MATLAB program (see the Supporting Information) as in the experiment described above. The frequency of liquid meniscus oscillation was calculated based on the variation in black pixels in each image using the fast Fourier transform (FFT). Additionally, the frequency of the ESI spray current was computed using FFT based on the signal recorded by the oscilloscope (.csv file exported from the OWON software; version 2.2.2; OWON Technology, Zhangzhou, China).

## Results and Discussion

### Correlation of MS Signals with the Formation of Taylor Cone by Offline Image Analysis

**Figure 3:**
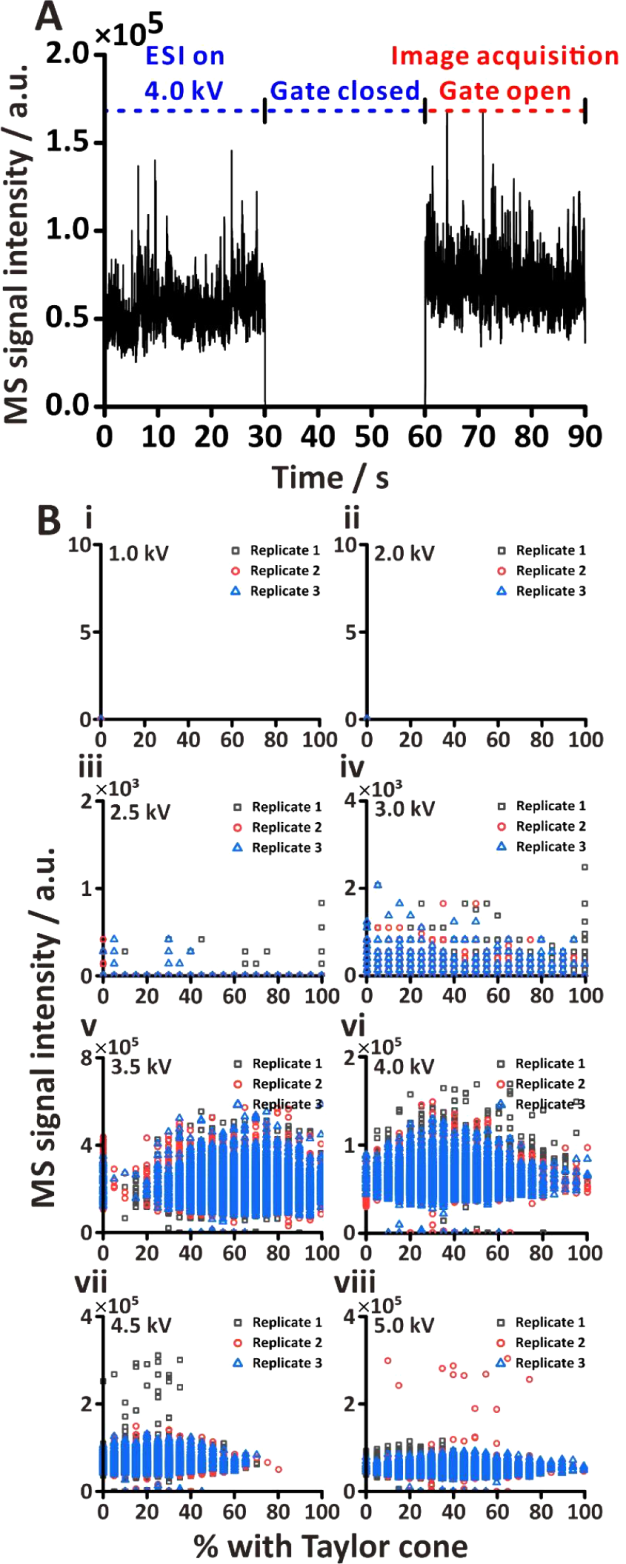
Correlation between MS signals and Taylor cone occurrence by offline image analysis. (A) MS ion current for correlating MS signals with the formation of the Taylor cone by offline image analysis. (B) Relationships between the percentage of Taylor cone time and MS signal intensities under varying ESI voltages: (i) 1.0 kV; (ii) 2.0 kV; (iii) 2.5 kV; (iv) 3.0 kV; (v) 3.5 kV; (vi) 4.0 kV; (vii) 4.5 kV; and (viii) 5.0 kV. The figure presents three replicates performed on day 1. A 20 μM acetaminophen solution dissolved in 25% (v/v) methanol in water was used as the sample and infused at a constant flow rate of 15 μL min^–1^. Data acquisition was performed using a QqQ-MS instrument operating in SIM mode at *m*/*z* 152. The frame rate was set at 5000 fps, capturing 150 000 frames over 30 s, with the Taylor cone percentage calculated every 20 frames.

In the first experiment, the relationship between the Taylor cone occurrence and MS ion current was revealed by offline image analysis. This experiment involved a plume gate (RE3) opening (30 s) to verify the relationship between the formation of the Taylor cone and the MS signals. For that purpose, we simultaneously acquired images and MS ion current data. A solution of 20 μM acetaminophen dissolved in 25% (v/v) MeOH in MS-grade water was used as the test sample. ESI voltages ranging from 0 to 5 kV were tested. In a typical experiment, the REs were supplied with the following voltages: RE1, 0 V; RE2, 300 V; RE3 and square wave from 0 to 1.65 kV. Figure 3A shows an example of a recorded ion current with an indicated image acquisition interval. The plume gate (RE3) was open during the time intervals 0–30 s and 60–90 s. The purpose of applying this time sequence was to enable synchronization between the start of MS signal registration and visual monitoring of the Taylor cone by the high-speed camera. Furthermore, we introduced time shifts to compensate for potential delays between image capturing and MS acquisition. Such shifts are rationalized by droplet and ion migration from the ion source, through the vacuum interface, the mass analyzers, to the detector. Spearman’s correlation coefficients (ρ) for datasets relating signal intensity with the percentage of Taylor cone occurrence were calculated. The results of this experiment (nine replicates in total, three replicates on each of three days) are shown in Figures 3B and S3–S7.

As expected, no signals were recorded for voltages of 0–2 kV (Figure 3Bi–Bii). Sporadic spike signals were recorded at 2.5 kV (Figure 3Biii). This voltage is close to the electrospray threshold voltage (here, 2.22 kV),^41,67^ which resulted in an unstable cone-jet. When the ESI voltage was increased to 3–5 kV, MS signals were recorded consistently for different percentages of Taylor cone formation per analysis time interval (Figure 3Biv–Bviii). Regardless of parameter settings (ESI voltage, time shift), all Spearman’s correlation (ρ) values were above −0.2 and below 0.2 (Figure S3), suggesting no correlation between the MS signals and the presence of the Taylor cone.

The absence of positive correlations in the current setup using a triple quadrupole mass spectrometer suggests the possibility of ion carryover on the way from the ion source to the detector. Overall, the apparent carryover can be due to the fluidic dynamics in the inlet, repulsive diffusion of the charged droplet and ions, and collisional diffusion inside the RF ion guide. Ions generated under atmospheric pressure undergo focusing before entering the quadrupole rods via the lens system, which includes the Q array, skimmer, multipole, and entrance lens (Figure S8).^68^ It is imaginable that ions are retained before their entry into the vacuum compartment and in the medium vacuum chamber, leading to the blending of the incoming ion packets on a millisecond time scale. Another limitation of the current setup may be the insufficient sampling rate of the mass spectrometer. Notably, the mass spectrometer only records ions for 0.8 ms, and most of the time (within the 4 ms event) is spent on data transfer. Moreover, the Taylor cone presence is sporadic in the 4 ms blocks. To harness Taylor cone pulsations for MS signal enhancement, it may be necessary to redesign the ion path from the ESI to the mass analyzer and use a rapid pulsed sampling mass analyzer.

### Real-Time Control for Synchronizing Taylor Cone Detection with MS Response

**Figure 4:**
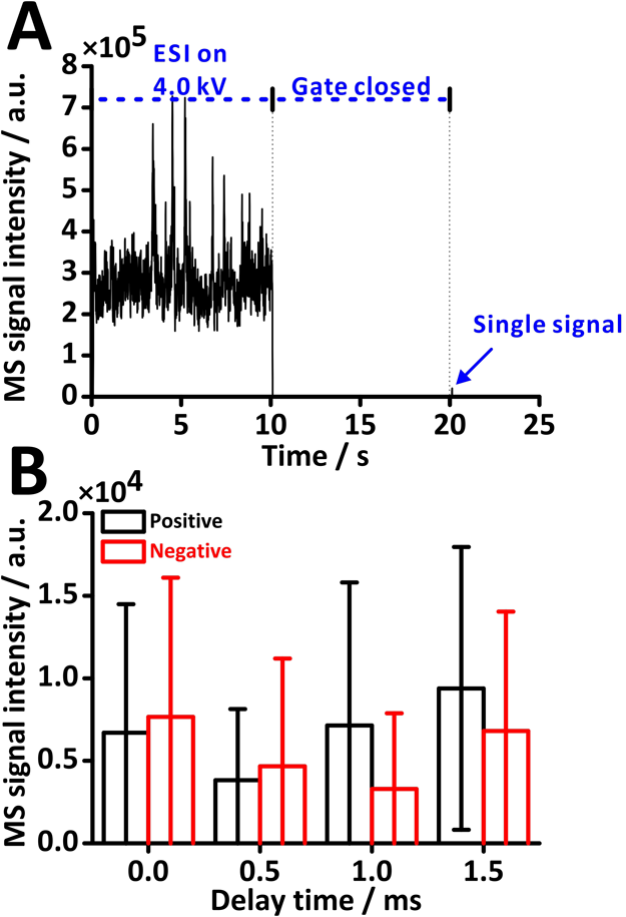
RTC system for synchronizing ESI-RE-MS with HSC. (A) A typical MS ion current was recorded in the experiment involving real-time control for synchronizing Taylor cone detection with MS response. (B) The relationship between average MS signal intensities and the set delay time before gate opening. Labels: positive—Taylor cone present; negative—Taylor cone absent. The gate opening duration was set to 1 ms. Camera frame rate: 5000 fps. Sample flow rate: 15 μL min^–1^. Sample: 20 μM acetaminophen in 25% (v/v) methanol in water. Data acquisition was conducted using the QqQ-MS instrument operated in SIM mode set to the *m*/*z* of 152.

To further investigate the relationship between Taylor cone formation and MS signal intensity and partly address the limitations of the previous experiment, a control system was utilized for real-time image analysis. This system effectively synchronizes image analysis results with the MS signal intensity by controlling the plume gate (RE3) under specific conditions corresponding to the presence or absence of the Taylor cone. When the Taylor cone was present, the condition was defined as “positive”, while its absence was defined as “negative”. To improve the synchronization, each run stopped immediately after the plume gate (RE3) closure (high potential), yielding a single signal per run (Figure 4A).

We verified the efficacy of the RTC algorithm. The ordinate in Figure S9 represents the time elapsed from image acquisition to gate opening, while the abscissa represents the delay time setting from image analysis completion to gate opening. Notably, both positive (*R*^2^ = 0.9895) and negative (*R*^2^ = 0.9978) RTC conditions exhibited relatively high *R*^2^ values. The time required for real-time computation was estimated to be ∼0.44 ms (*y*-intercept). This value is slightly smaller than the anticipated period of electrospray oscillations (0.5–1.0 ms for 1–2 kHz).^11,24,27^

Considering the droplet velocity, which takes approximately 1 ms to reach the plume gate (RE3),^32^ different delay times were tested to explore the relationship between the formation of the Taylor cone and MS signal intensities. Figure 4B compares positive and negative RTC values at various delay times. Despite the occurrence of some high peaks across the dataset, leading to substantial standard deviation in both positive and negative RTC values, the results show nearly identical intensities, suggesting that the formation of the Taylor cone does not significantly influence MS signal intensity. Irrespective of the delay time employed, the average intensities were similar regardless of the presence of the Taylor cone. However, this experiment has some limitations. For example, the lack of correlation can be affected by the experimental procedure, where a relatively long gate open time samples a range of Taylor cone states within a single electrospray pulsation cycle.

### Comparison of the Spray Currents Recorded by a Faraday Plate with High-Speed Imaging Datasets

Although the above results do not show a discernible correlation between the presence or absence of the Taylor cone and the MS ion currents, we conducted an offline experiment to verify the relationship between the frequency of the spray current and the frequency of the Taylor cone pulsation during the ESI process. A Faraday plate detector and oscilloscope were employed to record the spray current (Figure S2). Meanwhile, high-speed shadow imaging was utilized to capture images of oscillating Taylor cones in the same time interval. In the offline experiment, the presence of the Taylor cone was verified based on the total number of black pixels. The program returns the number of black pixels to represent the true oscillation. However, please note that—due to different settings in this experiment—the pixel number threshold used in the online experiment does not apply here. Using the total number of pixels—rather than a Boolean value—ensures that we capture the full dynamics of the Taylor cone’s behavior, including its collapse between pulses.

**Figure 5:**
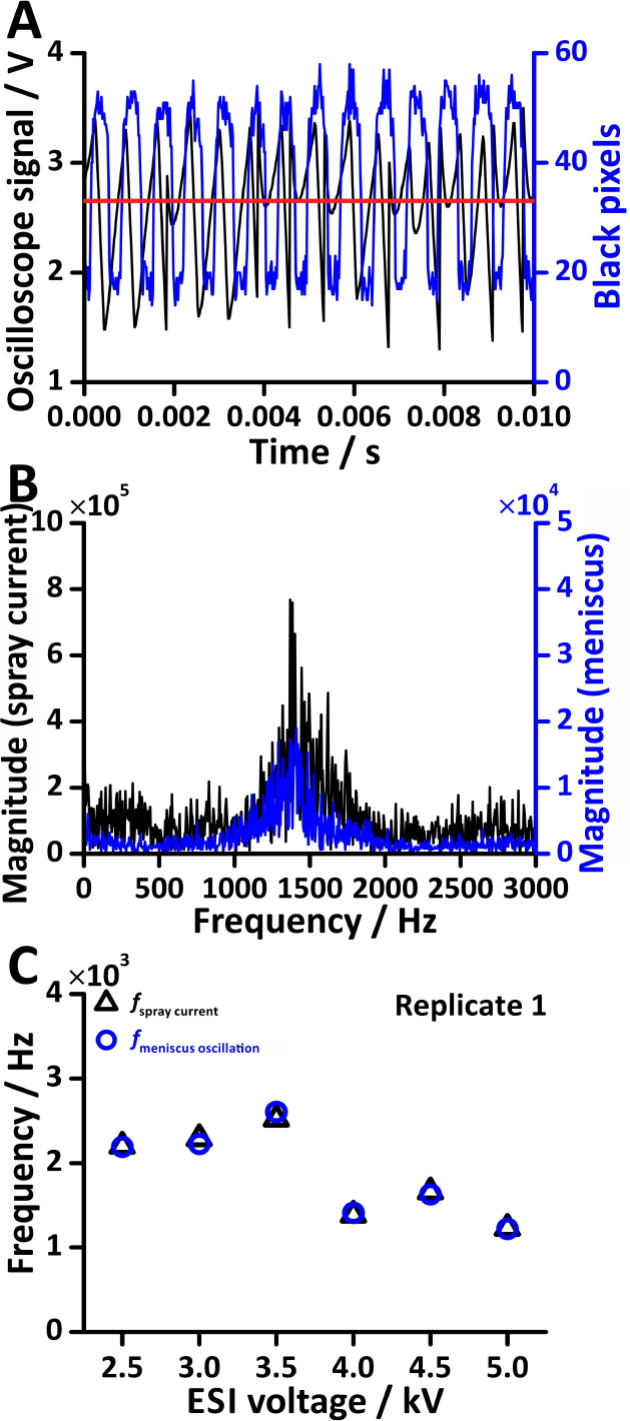
Simultaneous measurement of the liquid meniscus oscillation and the ESI spray current in offline experiments. (A) The raw data from the oscilloscope signal (black) and high-speed image analysis (blue) with ESI applied at 4.0 kV. The marked line indicates an arbitrary number of pixels above which the Taylor cone is considered to be present. (B) The FFT spectra from the oscilloscope signal (black) and high-speed image analysis (blue) with ESI applied at 4.0 kV. (C) The first replicate of the tests with varying ESI voltages. Sample flow rate: 15 μL min^–1^. Sample: 25% (v/v) methanol in water.

Notably, there is great similarity between the spray current frequency (∼1.93 kHz) and the liquid meniscus oscillation frequency (∼1.92 kHz; ESI voltage, 4.0 kV; cf. Figure 5A,B). Figure 5C depicts the spray current frequency (black triangles) and the frequency of liquid meniscus oscillation (blue circles) across various ESI voltages. Interestingly, around the apex of the black pixel peaks, the current varies significantly, while the black pixel counts stay relatively constant. Therefore, the image analysis may indicate the presence of a Taylor cone while actual ESI current and droplet emissions vary significantly. Further, the data trends across the three replicates exhibit remarkable consistency (Figure S10). This result is in agreement with the result reported by Marginean et al.^11^ who used refracted laser light for Taylor cone tracking. Interestingly, both frequencies decreased when the ESI voltage was increased from 3.5 kV to 4.0 kV. This behavior can be attributed to the transition in spraying modes caused by varying the ESI voltage. In fact, Nemes et al.^27^ previously investigated the influence of increasing ESI voltage. From 2.5 to 3.4 kV, the spraying mode of ESI changed from the burst mode (unstable current) to the pulsating Taylor cone mode (regular current oscillation) and finally to the cone-jet mode (stable Taylor cone formed without oscillation). The pulsating Taylor cone mode may transition to the cone-jet mode as the ESI voltage increases. This intermediate status may cause the oscillations to slow, resulting in a frequency drop between 3.5 and 4.0 kV.

## Conclusions

We have explored the relationship between electrospray Taylor cone formation and MS ion current intensity. Both offline image analysis and the RTC algorithm reveal no significant correlations across the varied ESI voltage settings. The MS signal intensity remains relatively stable at various delay times, regardless of the presence or absence of the Taylor cone, indicating minimal impact on the ion current. In an offline experiment, a Faraday plate was employed as a detector, revealing agreement between the frequency of liquid meniscus oscillation—followed by high-speed imaging—and the frequency of detected spray current. The lack of anticipated correlations between the formation of the Taylor cone and the MS signal can be explained by ion carryover along the ion path and insufficient MS sampling rate. A drawback of the current system is a relatively low time resolution and ion detection duty cycle of data acquisition within each MS event. Further explorations in this direction could employ mass analyzers with redesigned ion paths for rapid ion transfer and rapid submillisecond pulsed sampling. In the future, introducing such instruments and coupling them with the RTC system might potentially improve the sensitivity of MS methods.

## Supporting Information Available

Workflows of the RTC algorithm and the image analysis procedure in the online experiments; schematic diagram of the experimental setup involving spray current detection by Faraday plate; relationships between the percentage of Taylor cone time and MS signal intensities under varying ESI voltages; Spearman’s correlation coefficients for the relationships between MS signal intensities and the percentages of Taylor cone; ion path in the QqQ-MS instrument; image processing time of real-time analysis; spray current frequency and frequency of liquid meniscus oscillation across various ESI voltages; and computer code (PDF)
