# Supplementary material for: Does the Formation of a Taylor Cone in a Pulsating Electrospray Directly Impact Mass Spectrometry Signals?
Source: ACS Omega. 2024 Oct 11;9(42):43211–8. doi: 10.1021/acsomega.4c07653 (PMC11500155; doi:10.1021/acsomega.4c07653)
Supplement: Supplementary file 1 — ao4c07653_si_001.pdf [file ao4c07653_si_001.pdf]

SUPPORTING INFORMATION

Does the Formation of a Taylor Cone in a Pulsating Electrospray Directly Impact Mass Spectrometry Signals?

Ching-Han Chang, Pawel L. Urban*

Department of Chemistry, National Tsing Hua University

101, Section 2, Kuang-Fu Rd., Hsinchu, 300044, Taiwan

* Corresponding author:

P.L. Urban (urban@mx.nthu.edu.tw)

ADDITIONAL FIGURES

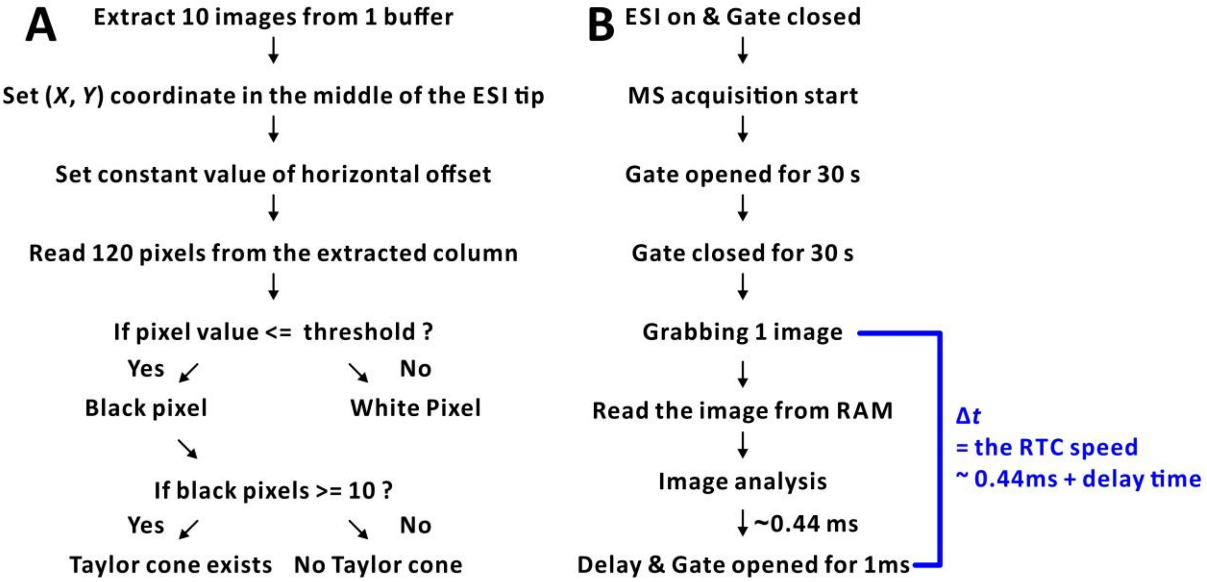

Figure S1. Workflows of (A) the RTC algorithm and (B) the image analysis procedure in the online experiments.

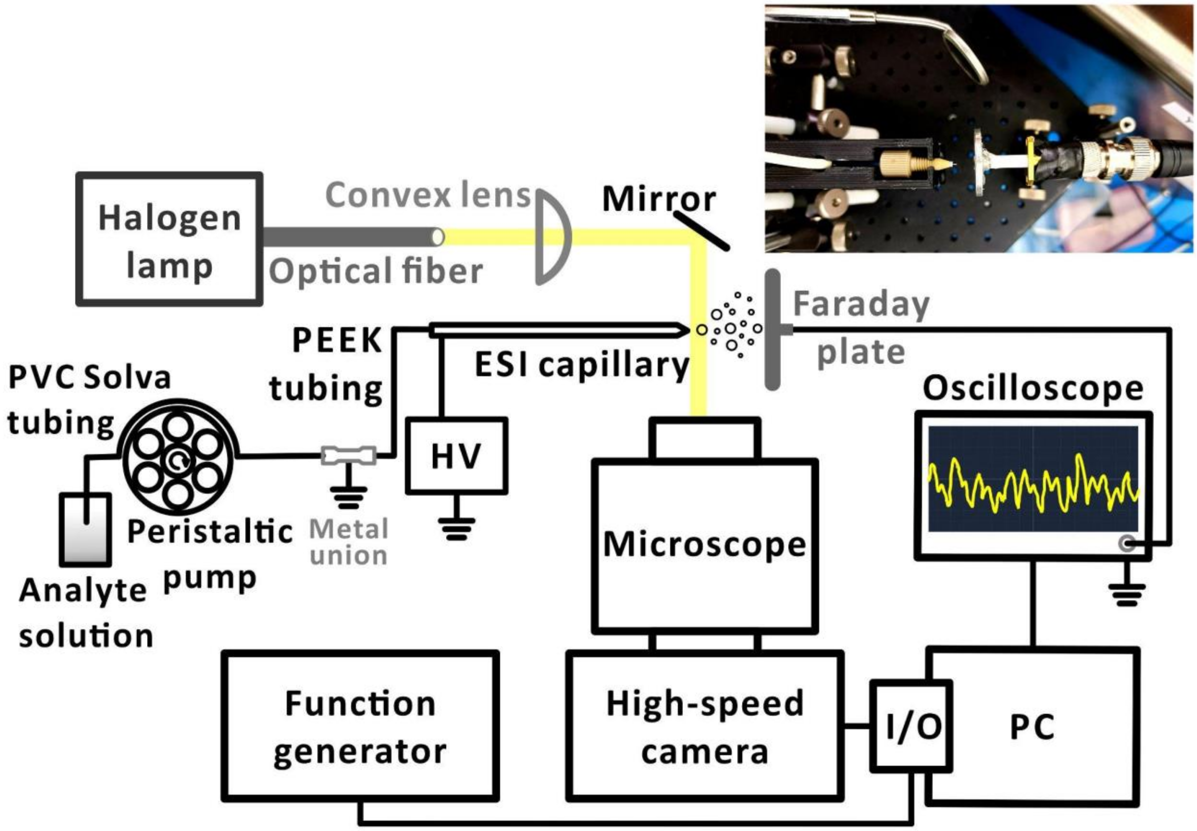

Figure S2. Schematic diagram of experimental setup involving spray current detection by Faraday plate.

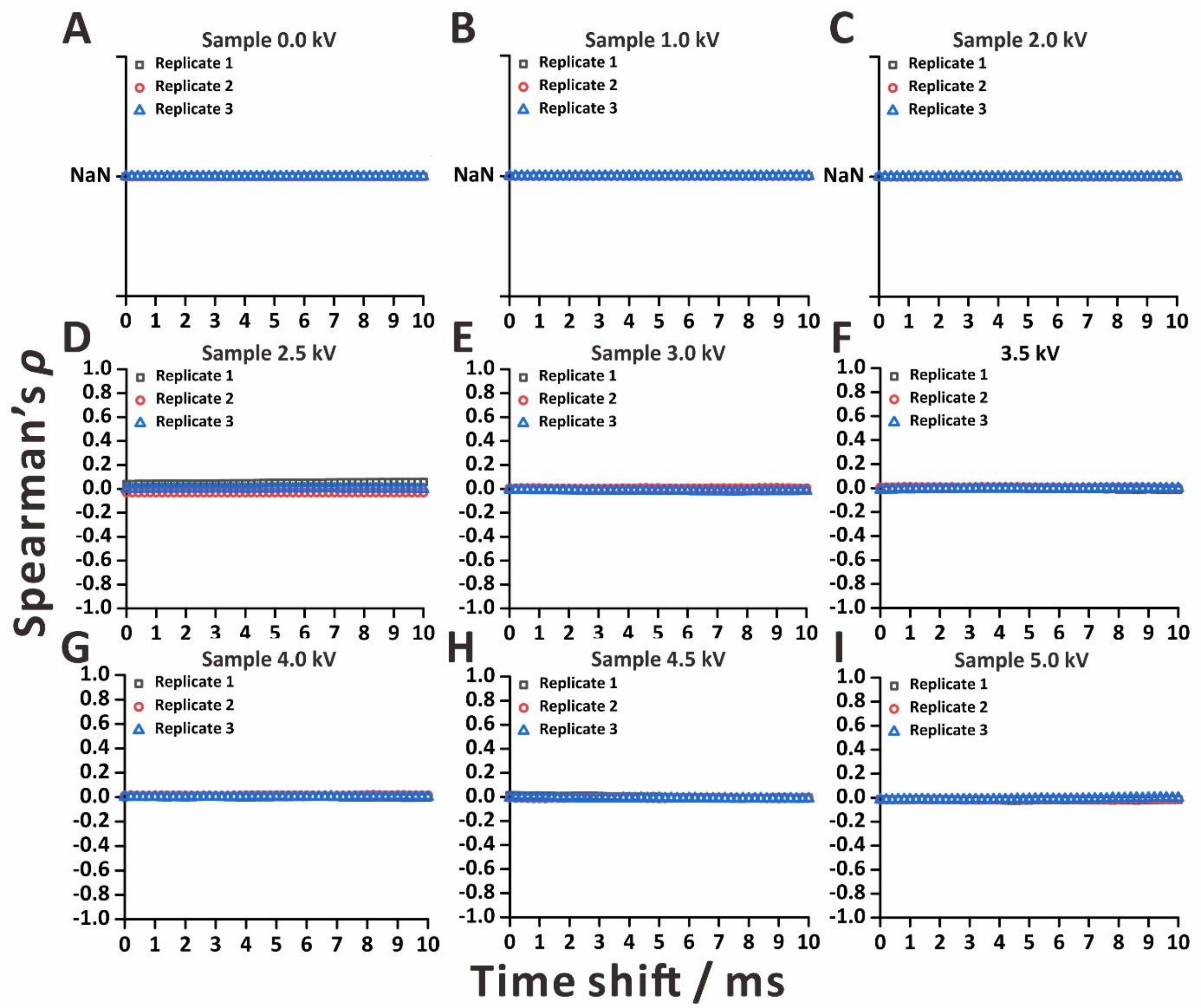

Figure S3. Spearman's correlation coefficients for the relationships between MS signal intensities and the percentages of Taylor cone were determined while taking into account the time shift (by adjusting the image range in image analysis). The time shift was incremented by 0.2 ms, corresponding to the frame rate of 5,000 fps. This figure presents the data from 3 replicates conducted on **day 1**.

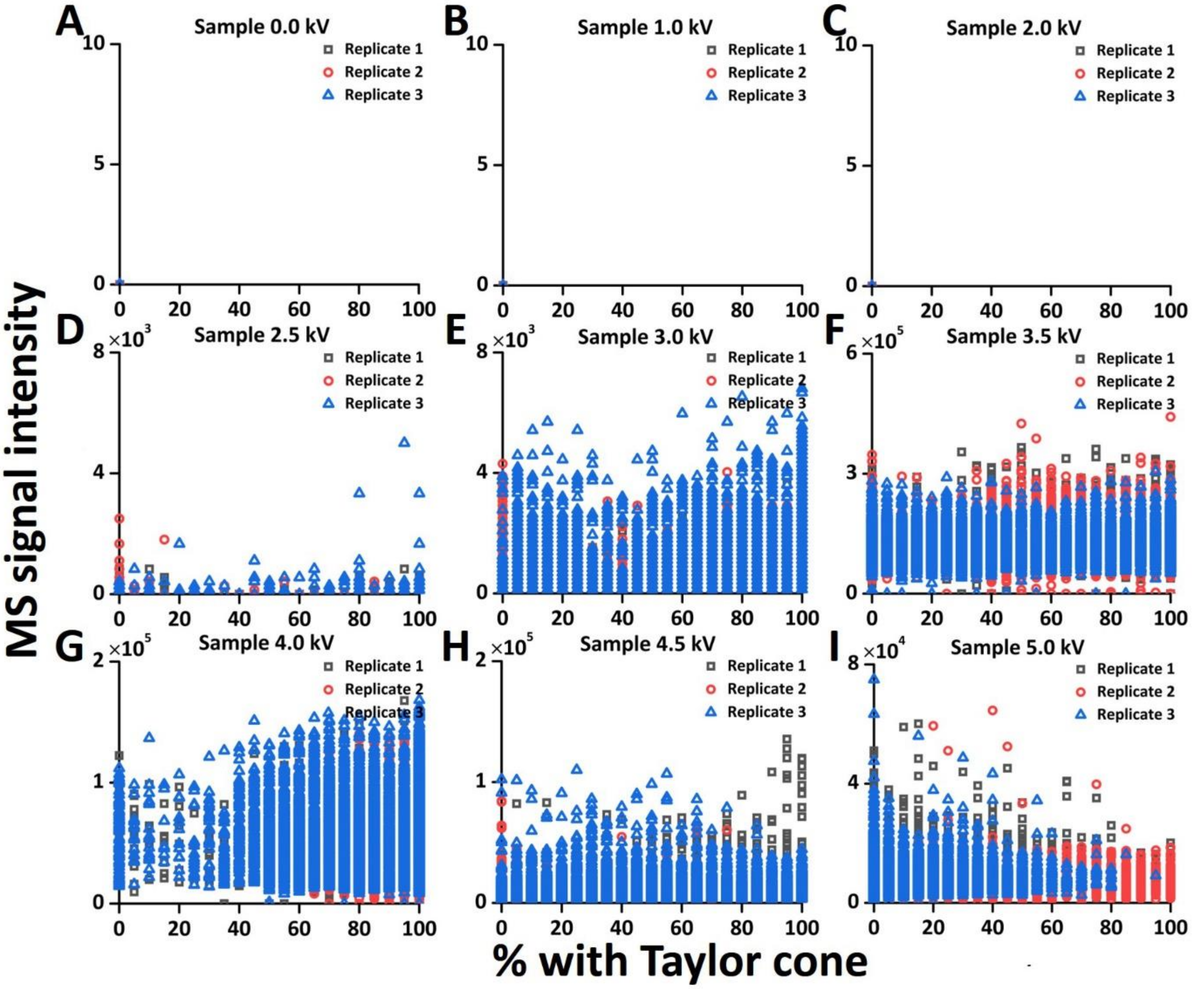

Figure S4. Relationships between the percentage of Taylor cone time and MS signal intensities under varying ESI voltages: (A) 0.0 kV; (B) 1.0 kV; (C) 2.0 kV; (D) 2.5 kV; (E) 3.0 kV; (F) 3.5 kV; (G) 4.0 kV; (H) 4.5 kV; and (I) 5.0 kV. The figure presents 3 replicates performed on **day 2**. 20 µM acetaminophen dissolved in 25% (v/v) methanol in water was used as the sample, and infused at a constant flow rate of 15 µL min^{-1}. Data acquisition was performed using a QqQ-MS operating in SIM mode at the *m/z* 152. The frame rate was set at 5000 fps, capturing 150,000 frames over 30 s, with the Taylor cone percentage calculated every 20 frames.

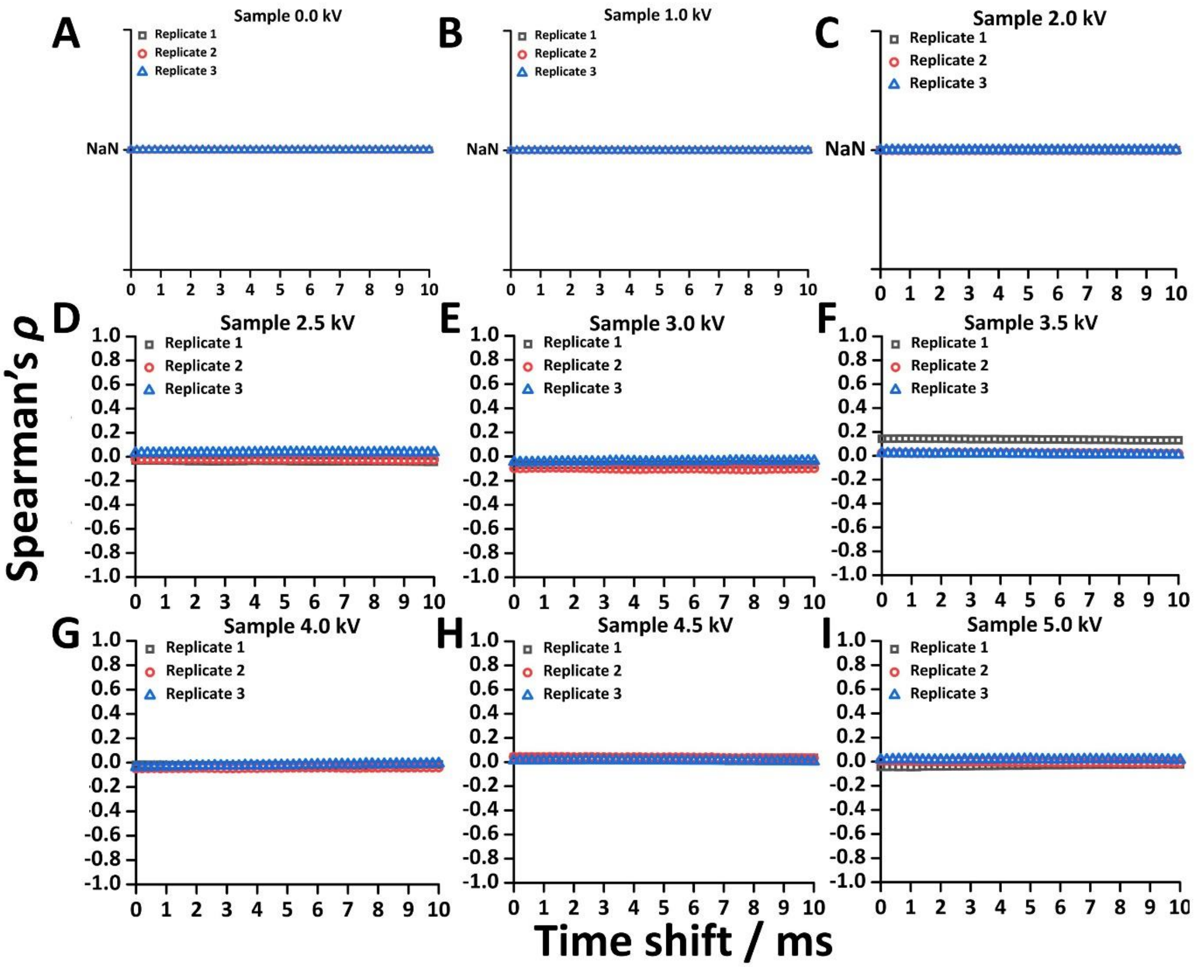

Figure S5. Spearman's correlation coefficients for the relationships between MS signal intensities and the percentages of Taylor cone were determined while taking into account the time shift (by adjusting the image range in image analysis). The time shift was incremented by 0.2 ms, corresponding to the frame rate of 5,000 fps. This figure presents the data from 3 replicates conducted on **day 2**.

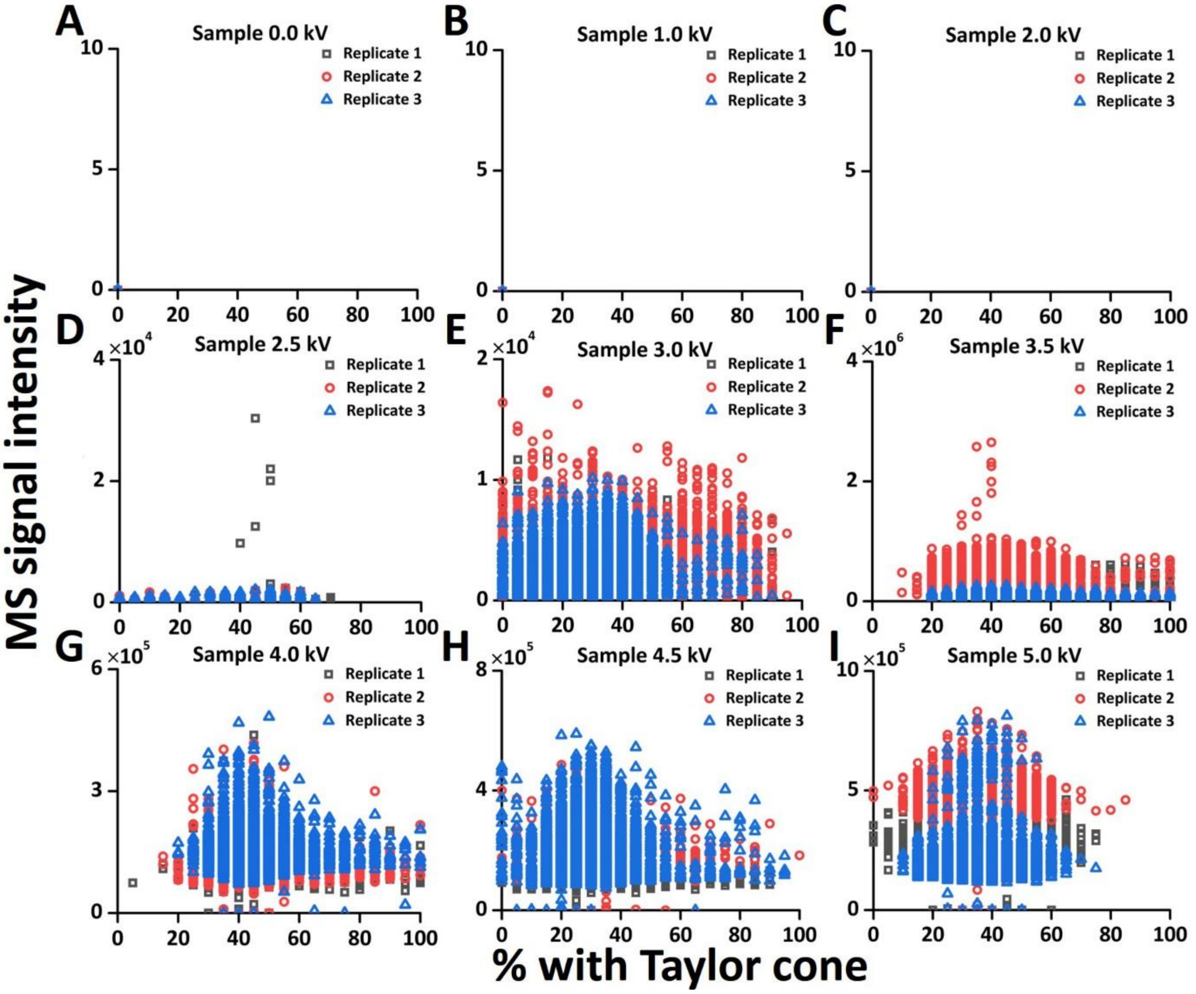

Figure S6. Relationships between the percentage of Taylor cone time and MS signal intensities under varying ESI voltages: (A) 0.0 kV; (B) 1.0 kV; (C) 2.0 kV; (D) 2.5 kV; (E) 3.0 kV; (F) 3.5 kV; (G) 4.0 kV; (H) 4.5 kV; and (I) 5.0 kV. The figure presents 3 replicates performed on **day 3**. 20 µM acetaminophen dissolved in 25% (v/v) methanol in water was used as the sample, and infused at a constant flow rate of 15 µL min^{-1}. Data acquisition was performed using a QqQ-MS operating in SIM mode at the *m/z* 152. The frame rate was set at 5000 fps, capturing 150,000 frames over 30 s, with the Taylor cone percentage calculated every 20 frames.

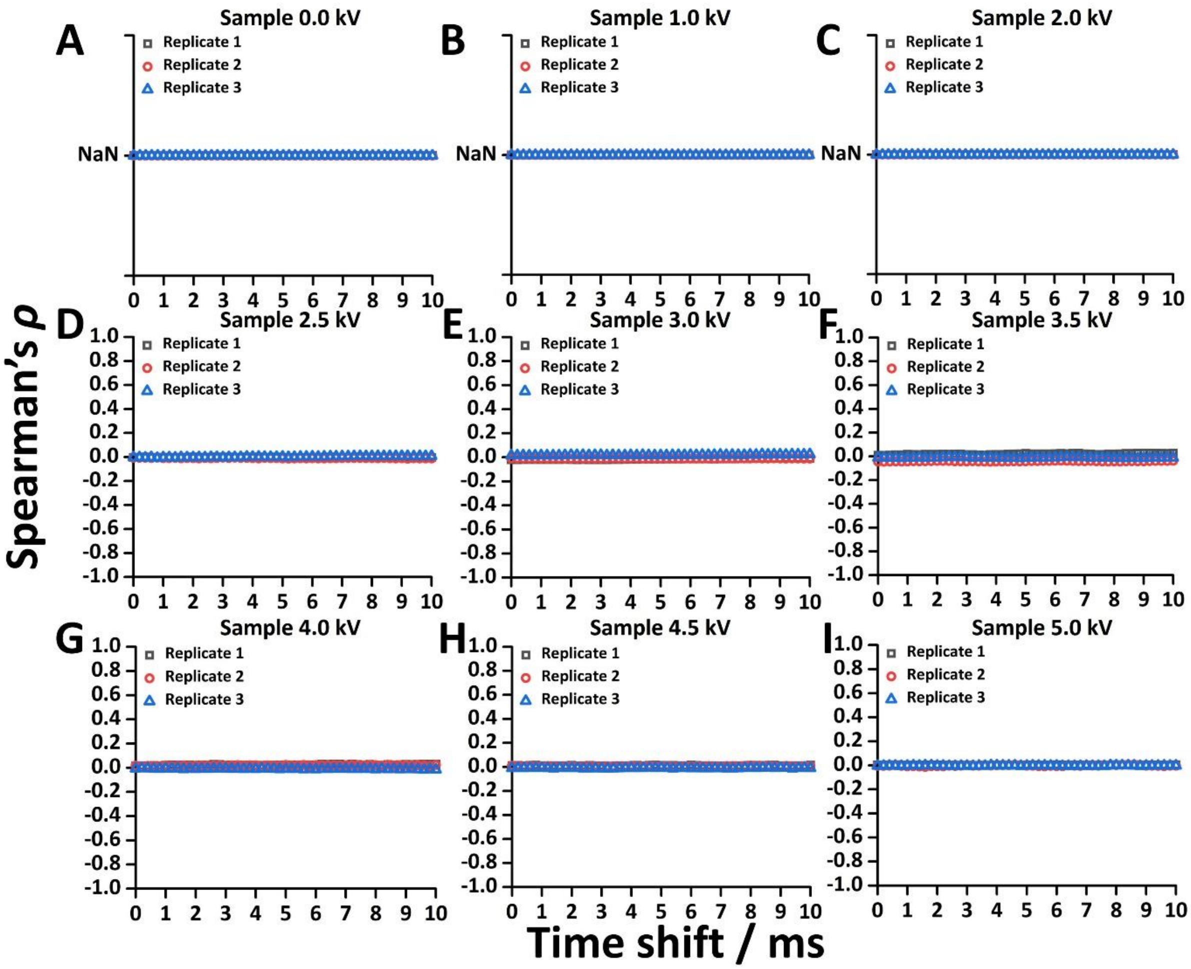

Figure S7. Spearman's correlation coefficients for the relationships between MS signal intensities and the percentages of Taylor cone were determined while taking into account the time shift (by adjusting the image range in image analysis). The time shift was incremented by 0.2 ms, corresponding to the frame rate of 5,000 fps. This figure presents the data from 3 replicates conducted on **day 3**.

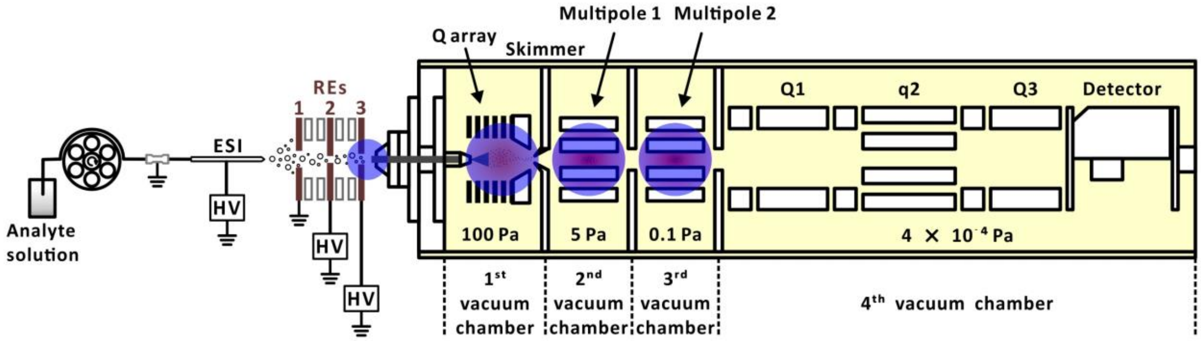

Figure S8. Ion path in the QqQ-MS instrument. The colored zones represent regions potentially causing ion retention.

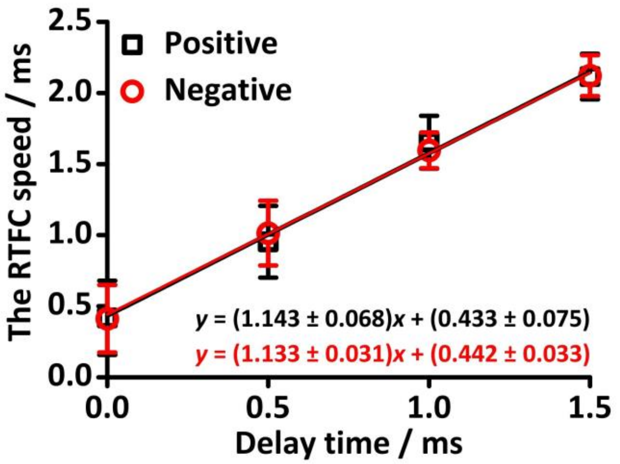

Figure S9. Image processing time of real-time analysis. R^2 is 0.9895 for positive (Taylor cone present) and 0.9978 for negative (Taylor cone not present) control.

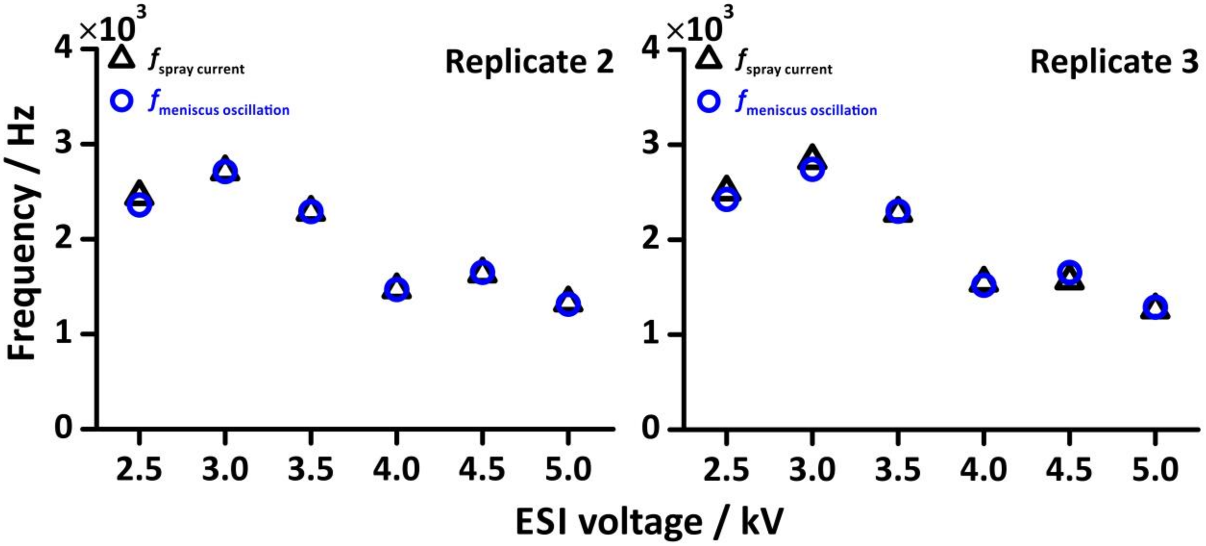

Figure S10. Spray current frequency (black triangles) and frequency of liquid meniscus oscillation (blue circles) across various ESI voltages.

COMPUTER CODE

Image processing for extracting images from raw buffer (Python)

```
import os
import cv2
import tkinter as tk
from tkinter import filedialog as fd
import time

# Graphical user interface
top = tk.Tk()
top.title('High speed camera images cropping')

frame = tk.Frame(top)
frame.pack()
space = tk.Label(frame, text = "        ", width = 40, height = 5)
space.pack()

def getFolder():
    top.dir = fd.askdirectory()
space = tk.Button(frame, text = "Select Folder", command = getFolder)
space.pack(side = tk.TOP)

frame = tk.Frame(top)
frame.pack()
space = tk.Label(frame, text = "        ", width = 40, height = 5)
space.pack()

frame = tk.Frame(top)
frame.pack()
space = tk.Button(frame, text = "OK", command = top.destroy)
space.pack(side = tk.TOP)

frame = tk.Frame(top)
frame.pack()
space = tk.Label(frame, text = "        ", width = 40, height = 5)
space.pack()

top.mainloop()

# Start Counting
count = 1
# Cropping images
Folder = top.dir

for i, filename in enumerate(os.listdir(Folder)):
    # Rename file
    Folder = top.dir
    new_name = 'origin'
```

```
    renamed_file = new_name + str(i) + ".jpeg"
    source_file = Folder + "/" + filename
    renamed_file = Folder + "/" + renamed_file
    os.rename(source_file, renamed_file)

    # Import images
    img = cv2.imread(Folder + '/origin' + str(i) + '.jpeg',0)

    buffercountnum = 100
    resolution_x = 256
    resolution_y = 256

    for j in range(0, buffercountnum):

        # Position to crop the images
        x = 0
        y = resolution_y * j

        # Width and height to crop
        w = resolution_x
        h = resolution_y

        # Crop images
        crop_img = img[y:y+h, x:x+w]

        # Save the processed image
        cv2.imwrite(Folder + '/CroppedImg.' + str(count).zfill(6) + '.jpg',
crop_img)
        count += 1

    # Convert original image from jpeg to jpg format
    cv2.imwrite(Folder + '/origin' + str(i) + '.jpg', img)
    os.remove(Folder + '/origin' + str(i) + '.jpeg')

# Task completion message
message = tk.Tk()
message.title('High speed camera images cropping')
frame4 = tk.Frame(message)
frame4.pack()
Text = tk.Label(frame4, text = "High speed camera images cropping
successful.", width = 50, height = 10)
Text.pack()
message.mainloop()

# References:
'''1. OpenCV - Adaptive Thresholding,
https://docs.opencv.org/master/d7/d4d/tutorial_py_thresholding.html
       2. OpenCV - Miscellaneous Image Transformations,
https://docs.opencv.org/master/d7/d1b/group__imgproc__misc.html
       3. OpenCV - Flags used for image file reading and writing,
https://docs.opencv.org/3.4/d8/d6a/group__imgcodecs__flags.html'''
```

Determining the formation of Taylor cone by the image analysis (MATLAB)

```
clear; close; clc;
folder = "C:\Users\PULab\desktop\ESI_voltages_Rep1";
files = dir(fullfile(folder, '*.jpg'));
path = fullfile({files.folder}, {files.name});
Data = zeros(150000, 1); % Create a empty matrix for 150000 frames
middleX = 65;
middleY = 130;
cropColumn = middleX + 50;
%in front of ESI tip 40 pixels (5.0 kV)
%in front of ESI tip 45 pixels (4.5 kV)
%in front of ESI tip 40 pixels (4.0 kV)
%in front of ESI tip 30 pixels (3.5 kV)
%in front of ESI tip 45 pixels (3.0 kV)
%in front of ESI tip 50 pixels (2.5 kV)
cropHi = middleY - 60;
cropHf = middleY + 60;
DataIndex = 1;
blackPixels = 0;
threshold = 30;

for i = 1:numel(path)
    img = imread(path{i});
    if size(img) == [256, 256]
        for y = cropHi:cropHf
                pixelValue = img(y, cropColumn); % Get pixel value (x, y)
                % Count black pixels
                if pixelValue < threshold
                  blackPixels = blackPixels + 1; % see as black
                end
        end
        if blackPixels > 10
             Data(DataIndex, 1) = 1; % Taylor cone existed
        else
             Data(DataIndex, 1) = 0; % No Taylor cone
        end
    else
        break;
    end
    blackPixels = 0;
    DataIndex = DataIndex + 1;
end
```

Counting percentage based on the formation of Taylor cone with time shift (MATLAB)

```
clc;close;clear;
file = 'C:\Users\PU Lab\Desktop\TaylorConeOnset.xlsx';

  range = 'A2:A150001'; % 5.0 kV
% range = 'B2:B150001'; % 4.5 kV
% range = 'C2:C150001'; % 4.0 kV
% range = 'D2:D150001'; % 3.5 kV
% range = 'E2:E150001'; % 3.0 kV
% range = 'F2:F150001'; % 2.5 kV

rep1 = readmatrix(file,'Sheet','rep1','Range', range);
rep2 = readmatrix(file,'Sheet','rep2','Range', range);
rep3 = readmatrix(file,'Sheet','rep3','Range', range);

numShifts = 51;
dataPointsPerShift = 7500; %5000fps, MS RUN 30s, event time 4 ms

Percentage1 = zeros(dataPointsPerShift,numShifts);
Percentage2 = zeros(dataPointsPerShift,numShifts);
Percentage3 = zeros(dataPointsPerShift,numShifts);


% Shift 1 data point per run, 51 runs in total
for shift = 1:numShifts
    % Calculate Taylor cone percentage by every 20 data points
    for i = 1:dataPointsPerShift
        startIndex = (i - 1) * 20 - shift +2;
        endIndex = startIndex + 19;

        if startIndex < 1
            % skip calculation & record a watermark '666'
            Percentage1(i, shift) = 666;
            Percentage2(i, shift) = 666;
            Percentage3(i, shift) = 666;
            continue;
        end

        if endIndex <= 150000
            sum1 = sum(rep1(startIndex:endIndex));
            sum2 = sum(rep2(startIndex:endIndex));
            sum3 = sum(rep3(startIndex:endIndex));
            Percentage1(i, shift) = sum1 * 100 / 20;
            Percentage2(i, shift) = sum2 * 100 / 20;
            Percentage3(i, shift) = sum3 * 100 / 20;
        else
            % if endIndex exceeds data range, skip calculation
            break;
        end
    end
end
```

Calculation of Spearman correlation coefficients with time shift (MATLAB)

```
clc;close;clear;
file = 'C:\Users\PU Lab\Desktop\TaylorConePercentage.xlsx';
range = 'B2:BA7501';
rep1 = readmatrix(file,'Sheet','rep1','Range', range);
rep2 = readmatrix(file,'Sheet','rep2','Range', range);
rep3 = readmatrix(file,'Sheet','rep3','Range', range);
intensityColumn = 52; %the column of intensity in EXCEL file

points = 7500;
Coe1 = zeros(51,4);
Coe2 = zeros(51,4);
Coe3 = zeros(51,4);

for i = 1:51
    if i == 1
        x1 = rep1(1:points, i); x2 = rep2(1:points, i); x3 = rep3(1:points, 
i);
        y1 = rep1(1:points, intensityColumn);y2 = rep2(1:points, 
intensityColumn); y3 = rep3(1:points, intensityColumn);
    elseif i > 1 && i <= 21
        x1 = rep1(2:points, i); x2 = rep2(2:points, i); x3 = rep3(2:points, 
i);
        y1 = rep1(2:points, intensityColumn);y2 = rep2(2:points, 
intensityColumn); y3 = rep3(2:points, intensityColumn);
    elseif i > 21 && i <= 41
        x1 = rep1(3:points, i); x2 = rep2(3:points, i); x3 = rep3(3:points, 
i);
        y1 = rep1(3:points, intensityColumn);y2 = rep2(3:points, 
intensityColumn); y3 = rep3(3:points, intensityColumn);
    else
        x1 = rep1(4:points, i); x2 = rep2(4:points, i); x3 = rep3(4:points, 
i);
        y1 = rep1(4:points, intensityColumn);y2 = rep2(4:points, 
intensityColumn); y3 = rep3(4:points, intensityColumn);
    end
        [RHOs,PVALs] = corr(x1,y1,'Type','Spearman'); [TAU,PVALk] = 
corr(x1,y1,'Type','Kendall');
        Coe1(i,1) = RHOs; Coe1(i,2) = PVALs; Coe1(i,3) = TAU; Coe1(i,4) = 
PVALk;
        [RHOs,PVALs] = corr(x2,y2,'Type','Spearman'); [TAU,PVALk] = 
corr(x2,y2,'Type','Kendall');
        Coe2(i,1) = RHOs; Coe2(i,2) = PVALs; Coe2(i,3) = TAU; Coe2(i,4) = 
PVALk;
        [RHOs,PVALs] = corr(x3,y3,'Type','Spearman'); [TAU,PVALk] = 
corr(x3,y3,'Type','Kendall');
        Coe3(i,1) = RHOs; Coe3(i,2) = PVALs; Coe3(i,3) = TAU; Coe3(i,4) = 
PVALk;
end
```

Real-time control (C++)

```
#include <iostream>
#include <EGrabber.h>
#include <chrono>
#include <vector>
#include <fstream>
using namespace Euresys;

// global objects
Euresys::EGenTL gentl;
Euresys::EGrabber<CallbackOnDemand> grabber(gentl);
std::vector< std::vector<uint64_t> > results; // use a vector to store IO
status
std::vector<uint64_t> rowData; //IO status of each analysis

// global variables
// set extract column & row
int middleX = 70;
int middleY = 115;
int cropWi = middleX + 40;
/*
in front of ESI tip 40 pixels (5.0 kV)
in front of ESI tip 45 pixels (4.5 kV)
in front of ESI tip 40 pixels (4.0 kV)
in front of ESI tip 30 pixels (3.5 kV)
in front of ESI tip 45 pixels (3.0 kV)
in front of ESI tip 50 pixels (2.5 kV)
*/
int cropHi = middleY - 60;
int cropWf = cropWi + 1;
int cropHf = middleY + 60;
// pixel analysis
uint64_t tsSTART = 0;
uint64_t tsREoff = 0;
uint64_t tsREon = 0;
uint64_t ts = 0;
uint64_t blackPixels = 0;
int threshold = 20;
uint64_t open = 0;
uint64_t close = 1;
uint64_t startMark = 666;

void delay_us(int timeout_us)
{
    auto start = std::chrono::high_resolution_clock::now();
    while (true)
    {
        if
(std::chrono::duration_cast<std::chrono::microseconds>(std::chrono::high_re
solution_clock::now() - start).count() >= timeout_us){break;}
    }
}
```

```
void MStrigger() {
    grabber.setString<Euresys::InterfaceModule>("LineSelector", "TTLIO11");
//MS trigger
    grabber.setString<Euresys::InterfaceModule>("LineMode", "Output");
    grabber.setString<Euresys::InterfaceModule>("LineInverter", "False");
    grabber.setInteger<Euresys::RemoteModule>("Width", 256);
    grabber.setInteger<Euresys::RemoteModule>("Height", 256);
    grabber.setFloat<Euresys::RemoteModule>("AcquisitionFrameRate",
5000.00); //Adjust frame rate here!
    grabber.setFloat<Euresys::RemoteModule>("ExposureTime", 1.00);
    grabber.setFloat<Euresys::RemoteModule>("Gain", 1.50);
    grabber.setString<Euresys::StreamModule>("DmaEngineOptimization",
"LowMemoryUsage");
    grabber.setInteger<Euresys::StreamModule>("BufferPartCount", 1);

    grabber.reallocBuffers(1); // allocate buffers, there are 1 buffers
here
    grabber.start(1);
    grabber.setString<Euresys::InterfaceModule>("LineSource", "High");
    delay_us(100000); // wait 0.1 seconds
    grabber.setString<Euresys::InterfaceModule>("LineSource", "Low");
    Euresys::ScopedBuffer buffer(grabber);
    tsSTART = buffer.getInfo<uint64_t>(GenTL::BUFFER_INFO_TIMESTAMP); // in
microsecondsstd::cout << "MSstart" << ", timestamp: " << tsSTART << " us"
<< std::endl;
    std::cout << "MSstart" << ", timestamp: " << tsSTART << " us" <<
std::endl;
    rowData = { blackPixels, startMark, tsSTART };
    results.push_back(rowData);
}

void initialization()
{
    MStrigger();
    grabber.setString<Euresys::InterfaceModule>("LineSelector", "TTLIO12");
    grabber.setString<Euresys::InterfaceModule>("LineMode", "Output");
    grabber.setString<Euresys::InterfaceModule>("LineInverter", "False");
    grabber.reallocBuffers(1); // allocate buffers, there are 1 buffers
here
    grabber.start(1);
    grabber.setString<Euresys::InterfaceModule>("LineSource", "Low");
    // Retrieve image buffer information
    Euresys::ScopedBuffer buf(grabber);
    // Get buffer information
    tsREoff = buf.getInfo<uint64_t>(GenTL::BUFFER_INFO_TIMESTAMP); // in
microseconds
    std::cout << "RE3off" << ", timestamp: " << tsREoff << " us" <<
std::endl;
    rowData = { blackPixels, startMark, tsREoff };
    results.push_back(rowData);
    delay_us(10000000); // wait 10 seconds
    grabber.reallocBuffers(1); // allocate buffers, there are 1 buffers
here
```

```
    grabber.start(1);
    grabber.setString<Euresys::InterfaceModule>("LineSource", "High");
    // Retrieve image buffer information
    Euresys::ScopedBuffer buffer(grabber);
    // Get buffer information
    tsREon = buffer.getInfo<uint64_t>(GenTL::BUFFER_INFO_TIMESTAMP); // in
microseconds
    std::cout << "RE3on" << ", timestamp: " << tsREon << " us" <<
std::endl;
    rowData = { blackPixels, startMark, tsREon};
    results.push_back(rowData);
    delay_us(10000000); // wait 10 seconds
}

void grab() {
    grabber.reallocBuffers(1); // allocate buffers, there are 1 buffers
here
    for (int i = 0; i < 1000; i++) //after 1 min, collecting 1 datas
    {
        grabber.start(1);         // capture 1 buffer(image)
        // Retrieve image buffer information
        Euresys::ScopedBuffer buffer(grabber);
        // Get buffer information
        void* imgPtr = buffer.getInfo<void*>(GenTL::BUFFER_INFO_BASE); //
get buffer address
        // std::string
format(gentl.imageGetPixelFormat(buffer.getInfo<uint64_t>(GenTL::BUFFER_INF
O_PIXELFORMAT))); // get buffer format
        size_t imgPitch =
buffer.getInfo<size_t>(GenTL::EuresysCustomGenTL::BUFFER_INFO_CUSTOM_LINE_P
ITCH);
        // Get buffer information
        ts = buffer.getInfo<uint64_t>(GenTL::BUFFER_INFO_TIMESTAMP); // in
microseconds
        // Assuming the image is in Mono8 format (8 bits per pixel)
        uint8_t* imageData = static_cast<uint8_t*>(imgPtr);
        // Access pixel values in the image
        for (size_t y = cropHi; y < cropHf; y++) {
            for (size_t x = cropWi; x < cropWf; x++) {
                uint8_t pixelValue = imageData[y * imgPitch + x]; // Get
the pixel value at (x, y)
                // Count black pixels
                if (pixelValue < threshold) {
                    blackPixels += 1; // see as black
                }
            }
        }
        // Positive feedback control
        if (blackPixels >= 10) { //with Taylor cone
            delay_us(1500);
            grabber.setString<Euresys::InterfaceModule>("LineSource",
"Low");
            delay_us(1000); // GOT 1.0 ms
```

```
            grabber.setString<Euresys::InterfaceModule>("LineSource",
"High");
            ts = buffer.getInfo<uint64_t>(GenTL::BUFFER_INFO_TIMESTAMP); //
in microseconds
            std::cout << "PositiveSFC" << ", timestamp: " << ts << " us" <<
std::endl;
            rowData = { blackPixels, open, ts };
            results.push_back(rowData);
            break;
        }

        // Negative feedback control
        if (blackPixels < 10) { //without Taylor cone
            delay_us(1500);
            grabber.setString<Euresys::InterfaceModule>("LineSource",
"Low");
            delay_us(1000); // GOT 1.0 ms
            grabber.setString<Euresys::InterfaceModule>("LineSource",
"High");
            ts = buffer.getInfo<uint64_t>(GenTL::BUFFER_INFO_TIMESTAMP); //
in microseconds
            std::cout << "NegativeSFC" << ", timestamp: " << ts << " us" <<
std::endl;
            rowData = { blackPixels, open, ts };
            results.push_back(rowData);
            break;
        }
        blackPixels = 0;
    }
}

int main()
{
    try
    {
        initialization();
        grab();
        grabber.setString<Euresys::InterfaceModule>("LineSource", "High");
        //write to csv file
        std::ofstream outputFile("C:/Users/PU Lab/Desktop/Data.csv");
        if (outputFile.is_open())
        {
            // write ‘header’
            outputFile << "Black Pixels, Status, Time Stamp\n";

            // write ‘data’
            for (const auto& row : results)
            {
                for (const auto& element : row)
                {
                    outputFile << element << ",";
                }
                outputFile << "\n";
```

```
            }
            outputFile.close();
        }
        else
        {
            std::cerr << "Unable to open output file." << std::endl;
        }
    }
    catch (const std::exception& e)
    {
        std::cout << "error: " << e.what() << std::endl;
    }
}
```

Converting spray current collected by Faraday plate to frequency domain by fast Fourier transform (MATLAB)

```
clc;clear;close;
%import excel data, file name, sheet name, range
FILE_off = 'C:/Users/PU Lab/Desktop/ESIoff.xlsx';
FILE_on = 'C:/Users/PU Lab/Desktop/ESIon.xlsx';
AMP = ', 5.0 kV';
ESI='5.0 kV';
rep = ', Rep3';
V = '25% MeOH';

t=readmatrix(FILE_off,'Range','C:C'); % TIME 'Sheet',SHEET,
Yoff=readmatrix(FILE_off,'Range','D:D'); % RESPONSE
Yon = readmatrix(FILE_on,'Range','D:D'); % RESPONSE
Tevent = 0.00002; % sampling rate, seconds

plot(t,Yoff,'linewidth',1,'Color','black') %draw raw data
hold on
plot(t,Yon,'linewidth',1,'Color','blue') %draw raw data
xlabel('Time / s')
ylabel('Response / mV')
title(['Raw: ',V,rep])
legend('0 kV',ESI,'Location','northwest')
axis([0 inf -inf inf]) %start from x=0 y=0
set(gca,"FontName","Calibri","FontSize",28,'FontWeight','bold')
hold off

y = fft(Yoff);
y2 = fft(Yon);
L=length(Yoff);
fs = 1/Tevent; % sampling frequency
f = fs/L*(0:L-1);

fshift = fs/L*(-L/2:L/2-1);
yshift = fftshift(y);
ABSY = abs(yshift);
yshift2 = fftshift(y2);
ABSY2 = abs(yshift2);

plot(fshift,ABSY,'linewidth',1,'Color','black');
hold on
plot(fshift,ABSY2,'linewidth',1,'Color','blue');
xlabel('Frequency / Hz')
ylabel('Magnitude')
axis([0 3000 0 inf])%start from x=0 y=0
legend('0 kV',ESI,'Location','northwest')
title(['FFT: ',V,rep])%5 μM HPF mixture at AC 200 Hz
set(gca,"FontName","Calibri","FontSize",28,'FontWeight','bold')
hold off
```

Extracting liquid meniscus oscillation information from images (MATLAB)

```
clc;clear;close;
folder = "C:/Users/PU Lab/Desktop/HSC_5.0kV_rep1";
files = dir(fullfile(folder, '*.jpg'));
path = fullfile({files.folder}, {files.name});
Data = zeros(10000, 1); % 10000 frames
middleX = 40;
middleY = 130;
cropColumn = middleX + 40;
%in front of ESI tip 40 pixels (5.0 kV) ~ 120 um
%in front of ESI tip 45 pixels (4.5 kV) ~ 135 um
%in front of ESI tip 25 pixels (4.0 kV) ~ 75 um
%in front of ESI tip 30 pixels (3.5 kV) ~ 90 um
%in front of ESI tip 45 pixels (3.0 kV) ~ 135 um
%in front of ESI tip 50 pixels (2.5 kV) ~ 150 um
cropHi = middleY - 60;
cropHf = middleY + 60;
DataIndex = 1;
blackPixels = 0;
threshold = 30;

for i = 1:numel(path)
    img = imread(path{i});
    if size(img) == [256, 256]
        for y = cropHi:cropHf
                pixelValue = img(y, cropColumn); % Get the pixel value at
(x, y)
                % Count black pixels
                if pixelValue < threshold
                  blackPixels = blackPixels + 1; % see as black
                end
        end
        Data(DataIndex, 1) = blackPixels; % with Taylor con
    else
        break;
    end
    blackPixels = 0;
    DataIndex = DataIndex + 1;
end
```

Converting liquid meniscus oscillations captured by HSC to frequency domain by fast Fourier transform (MATLAB)

```
clc;clear;close;
%import excel data, file name, sheet name, range
FILE = 'C:/Users/PU Lab/Desktop/HSC.xlsx';
AMP = ', 25% MeOH';
V = 'ESI 5.0 kV';
rep = ', Rep 3';

t=readmatrix(FILE,'Range','A:A'); % TIME 'Sheet',SHEET,
x=readmatrix(FILE,'Range','Y:Y'); % Black pixels
Tevent = 0.00002; % sampling rate, seconds

plot(t,x,'linewidth',1) %draw raw data
xlabel('Time / s')
ylabel('Pixel intensity')
title([V,', Raw:', AMP, rep])
axis([0 inf -inf inf]) %start from x=0 y=0
set(gca,"FontName","Calibri","FontSize",28,'FontWeight','bold')

y = fft(x);
n=length(x);
fs = 1/Tevent; % sampling frequency
f = (0:length(y)-1)*fs/length(y);

fshift = (-n/2:n/2-1)*(fs/n);
yshift = fftshift(y);
ABSY = abs(yshift);
plot(fshift,ABSY,'linewidth',1);
xlabel('Frequency / Hz')
ylabel('Magnitude')
axis([0 3000 0 inf])%start from x=0 y=0
title(['HSC, ',V,AMP,rep])%5 µM HPF mixture at AC 200 Hz
set(gca,"FontName","Calibri","FontSize",28,'FontWeight','bold')
X = transpose(fshift);
```

Circuit control for triggering the HV of RE3, the HSC, and QqQ-MS (Arduino)

```
int tri = 10;
int cam = 12;
int re = 8;
int but = 7;
int i;
int buttonstate = 0;

void setup() {
  pinMode(cam, OUTPUT);
  pinMode(re, OUTPUT);
  pinMode(tri, OUTPUT);
  pinMode(but, INPUT);
}

void loop() {
  digitalWrite(cam, LOW);
  digitalWrite(re, LOW);
  buttonstate = digitalRead(but);
  if (buttonstate == HIGH){
    digitalWrite(re, HIGH);
    digitalWrite(tri, HIGH);  // MS trigger
    delay(100);
    digitalWrite(tri, LOW);
    digitalWrite(re, LOW);
    delay(30000);
    digitalWrite(re, HIGH);
    delay(30000);
    digitalWrite(re, LOW);
    for (i = 0; i < 150000; i++){
        digitalWrite(cam, HIGH);
        delayMicroseconds(200);
        digitalWrite(cam, LOW);
        delayMicroseconds(200);
    }
    }
}
```

Circuit control for recording the status of TTLIO11 (MS trigger) and TTLIO12 (the HV of RE3) to calculate the RTC speed (Arduino)

```
int TTLIO11 = 2; int TTLIO12 = 3; int Time = 0; int i = 0;
int last11 = 0; int last12 = 1; int state11 = 0; int state12 = 1;
void setup() {
  pinMode(TTLIO11, INPUT);
  pinMode(TTLIO12, INPUT);
  Serial.begin(115200);
}
void loop() {
  state11 = digitalRead(TTLIO11);
  state12 = digitalRead(TTLIO12);
  Serial.print(“Time, Pin, Status\n”);

  while(true){
    state11 = digitalRead(TTLIO11);
    state12 = digitalRead(TTLIO12);
    if(state11 != last11 && state12 != last 12{
      Time = micros();
      Serial.print(Time);
      Serial.print(“, ”);
      Serial.print(11);
      Serial.print(“, ”);
      Serial.print(state11);
      Serial.print(“, ”);
      Serial.print(12);
      Serial.print(“, ”);
      Serial.print(state12);
      Serial.print(‘\n’);
      last11 = state11; last12 = state12;
    }
    else if(state11 == last11 && state12 != last 12{
      Time = micros();
      Serial.print(Time);
      Serial.print(“, ”);
      Serial.print(12);
      Serial.print(“, ”);
      Serial.print(state12);
      Serial.print(‘\n’);
      last12 = state12;
    }
    else if(state11 != last11 && state12 == last 12{
      Time = micros();
      Serial.print(Time);
      Serial.print(“, ”);
      Serial.print(11);
      Serial.print(“, ”);
      Serial.print(state11);
      Serial.print(‘\n’);
      last11 = state11;
    }
  }
```
